# Draft Genome Sequence of NDM-5-Producing Escherichia coli Sequence Type 648 and Genetic Context of *bla*_NDM-5_ in Australia

**DOI:** 10.1128/genomeA.00194-15

**Published:** 2015-04-09

**Authors:** Alexander M. Wailan, David L. Paterson, Michael Caffery, David Sowden, Hanna E. Sidjabat

**Affiliations:** a The University of Queensland, UQ Centre for Clinical Research, Queensland, Australia; b Pathology Queensland, Queensland, Australia

## Abstract

We report here the draft genome sequence of uropathogenic Escherichia coli sequence type 648 (ST648) possessing *bla*_NDM-5_ from a 55-year-old female in Australia with a history of travel to India. The plasmid-mediated *bla*_NDM-5_ was in a genetic context nearly identical to that of the GenBank entry of an IncX3 *bla*_NDM-5_ plasmid previously reported from India (Klebsiella pneumoniae MGR-K194).

## GENOME ANNOUNCEMENT

The Indian subcontinent has been reported as a geographical reservoir for the acquisition of NDM-producing *Enterobacteriaceae* (1). A 55-year-old female with chronic diarrhea had carbapenem-resistant Escherichia coli isolated from a urine sample in January 2014. She traveled to India in late 2013 and developed diarrhea but was not admitted to a medical facility. Upon her return to Australia, ongoing diarrhea prompted multiple hospital admissions. She was diagnosed with Crohn’s disease. During admission, a midstream urine sample was collected, from which the carbapenem-resistant E. coli CR694 was identified.

Whole-genomic DNA of E. coli CR694 was prepared using the Nextera XT DNA sample preparation kit (Illumina, USA) and sequenced using the Illumina HiSeq 2000 (Illumina) at the Australian Genome Research Facility. *De novo* assembly was performed using CLC Genomics Workbench version 7.5 (CLC bio, Denmark). The draft genome consists of 5,523,407 bp. The contigs were initially annotated using RAST (http://rast.nmpdr.org/). A BLAST analysis and manual annotation utilized previously reannotated reference sequences and IS Finder (https://www-is.biotoul.fr/). The MLST, ResFinder, and PlasmidFinder (http://www.genomicepidemiology.org/) databases were used to characterize sequence typing (ST), antibiotic resistance mechanisms, and the plasmid Inc types, respectively, of E. coli CR694. ST648, plasmid Inc types of IncFII, IncFIB, IncX3, IncI1, and IncX4, and the genes *bla*_NDM-5_, *bla*_CMY-42_, *aac-6-Ib-cr*, *aadA5*, *erm*(B), *mph*(A), *sul1*, *tet*(B), and *dfrA17* were identified.

Additionally, the annotation through RAST identified the type 1 fimbriae genes *fimABCDEFGHI*, virulence determinants relevant for urinary tract adhesion (2). Further, five other types of fimbriae were identified as a membrane transport type VII protein secretion system, namely the (i) *htrE* fimbriae cluster, (ii) *stf* fimbriae cluster, (iii) alpha-fimbriae, (iv) colonization factor antigen I fimbriae (CFA/I fimbriae), and (v) *sfm* fimbrial cluster. A cluster responsible for curli production or type VIII secretion was identified. Siderophore enterobactin, aerobactin, and other hemin transport systems for iron acquisition were identified. In addition, type IV pilus and an IncF conjugal transfer system were identified. A gene for serum survival (*iss*) was also identified. The identified virulence determinants may have contributed to the infection and or colonization of CR694 in the urinary tract (2).

The contig pCR694-EC-NDM-5 carried the *bla*_NDM-5_ genetic context. *bla*_NDM_ has been reported to reside within a 10,099-bp transposon known as Tn*125* (3). *bla*_NDM-5_ on pCR694-EC-NDM-5 was found to be located within a truncated 3,167 bp Tn*125* structure, flanked by an IS*5* upstream and an IS*26* downstream. pCR694-EC-NDM-5 was identical to an NDM-5 IncX3 plasmid, pNDM-MGR194 (as a direct submission, with GenBank accession no. KF220657) (4). Both *bla*_NDM-5_ genetic contexts did not possess the Tn*125*-associated genes *groES*, *groEL*, and IS*CR27*. Both pCR694-EC-NDM-5 and pNDM-MGR194 are also highly similar to NDM-1 IncX plasmid pKPN5047 (GenBank accession no. NC_020811), containing a longer Tn*125* structure in which *groES*, *groEL*, and IS*CR27* are present.

The *bla*_NDM-5_ genetic context of pCR694-EC-NDM-5 has not been reported within E. coli or within Australia. NDM-5-producing *Enterobacteriaceae* have been reported in Japan, Algeria, the United Kingdom, and India, of which an E. coli ST648 harbored *bla*_NDM-5_ in both the aforementioned United Kingdom and Japan reports (5–8). This case of an NDM-5-producing typical uropathogenic E. coli isolate highlights the further intercontinental acquisition of carbapenemase-producing *Enterobacteriaceae* through travel to geographical reservoirs.

This work was approved by the Royal Brisbane and Women's Human Research Ethics Committee (HREC/13/QRBW/391>: Epidemiology, clinical significance, treatment and outcome of infections by carbapenem-resistant *Enterobacteriaceae* and Acinetobacter spp. in Queensland).

### Nucleotide sequence accession numbers.

This project is registered as BioProject PRJNA268254 and BioSample SAMN03217331. The *bla*_NDM-5_ genetic context pCR694-EC-NDM-5 was submitted to the GenBank database and assigned the accession no. KP178355. The draft genome sequence of NDM-5-producing E. coli ST648 has been deposited in GenBank under accession no. JTGI00000000.

## References

[1] Wailan AM, Paterson DL 2014 The spread and acquisition of NDM-1: a multifactorial problem. Expert Rev Anti Infect Ther 12:91–115. doi:10.1586/14787210.2014.856756. 24308710

[2] Totsika M, Beatson SA, Sarkar S, Phan MD, Petty NK, Bachmann N, Szubert M, Sidjabat HE, Paterson DL, Upton M, Schembri MA 2011 Insights into a multidrug resistant *Escherichia coli* pathogen of the globally disseminated ST131 lineage: genome analysis and virulence mechanisms. PLoS One 6:e26578. doi:10.1371/journal.pone.0026578. 22053197 PMC3203889

[3] Hu H, Hu Y, Pan Y, Liang H, Wang H, Wang X, Hao Q, Yang X, Yang X, Xiao X, Luan C, Yang Y, Cui Y, Yang R, Gao GF, Song Y, Zhu B 2012 Novel plasmid and its variant harboring both a *bla*_NDM-1_ gene and type IV secretion system in clinical isolates of *Acinetobacter lwoffii*. Antimicrob Agents Chemother 56:1698–1702. doi:10.1128/AAC.06199-11. 22290961 PMC3318331

[4] Krishnaraju M, Kamatchi C, Jha AK, Devasena N, Vennila R, Sumathi G, Vaidyanathan R 2015 Complete sequencing of an IncX3 plasmid carrying *bla*_NDM-5_ allele reveals an early stage in the dissemination of the *bla*_NDM_ gene. Indian J Med Microbiol 33:30–38. doi:10.4103/0255-0857.148373. 25559999

[5] Nakano R, Nakano A, Hikosaka K, Kawakami S, Matsunaga N, Asahara M, Ishigaki S, Furukawa T, Suzuki M, Shibayama K, Ono Y 2014 First report of metallo-β-lactamase NDM-5-producing *Escherichia coli* in Japan. Antimicrob Agents Chemother 58:7611–7612. doi:10.1128/AAC.04265-14. 25246390 PMC4249512

[6] Sassi A, Loucif L, Gupta SK, Dekhil M, Chettibi H, Rolain JM 2014 NDM-5 carbapenemase-encoding gene in multidrug-resistant clinical isolates of *Escherichia coli* from Algeria. Antimicrob Agents Chemother 58:5606–5608. doi:10.1128/AAC.02818-13. 24982080 PMC4135848

[7] Rahman M, Shukla SK, Prasad KN, Ovejero CM, Pati BK, Tripathi A, Singh A, Srivastava AK, Gonzalez-Zorn B 2014 Prevalence and molecular characterisation of New Delhi metallo-beta-lactamases NDM-1, NDM-5, NDM-6 and NDM-7 in multidrug-resistant *Enterobacteriaceae* from India. Int J Antimicrob Agents 44:30–37. doi:10.1016/j.ijantimicag.2014.03.003. 24831713

[8] Hornsey M, Phee L, Wareham DW 2011 A novel variant, NDM-5, of the New Delhi metallo-β-lactamase in a multidrug-resistant *Escherichia coli* ST648 isolate recovered from a patient in the United Kingdom. Antimicrob Agents Chemother 55:5952–5954. doi:10.1128/AAC.05108-11. 21930874 PMC3232805

